# A protocol to extend the longitudinal coverage of on‐board cone‐beam CT

**DOI:** 10.1120/jacmp.v13i4.3796

**Published:** 2012-07-05

**Authors:** Dandan Zheng, Jun Lu, Ariel Jefferson, Cheng Zhang, Jian Wu, William Sleeman, Elisabeth Weiss, Nesrin Dogan, Shiyu Song, Jeffrey Williamson

**Affiliations:** ^1^ Department of Radiation Oncology University of Nebraska Medical Center Omaha NE; ^2^ Department of Radiation Oncology SUNY Upstate Medical University Syracuse NY; ^3^ Department of Radiation Oncology Virginia Commonwealth University Richmond VA; ^4^ Department of Electrical and Computer Engineering The University of Iowa Iowa City IO; ^5^ The University of Florida Proton Therapy Institute Jacksonville FL USA

**Keywords:** CBCT, field of view, IGART, image registration

## Abstract

The longitudinal coverage of a LINAC‐mounted CBCT scan is limited to the corresponding dimensional limits of its flat panel detector, which is often shorter than the length of the treatment field. These limits become apparent when fields are designed to encompass wide regions, as when providing nodal coverage. Therefore, we developed a novel protocol to acquire double orbit CBCT images using a commercial system, and combine the images to extend the longitudinal coverage for image‐guided adaptive radiotherapy (IGART). The protocol acquires two CBCT scans with a couch shift similar to the “step‐and‐shoot” cine CT acquisition, allowing a small longitudinal overlap of the two reconstructed volumes. An in‐house DICOM reading/writing software was developed to combine the two image sets into one. Three different approaches were explored to handle the possible misalignment between the two image subsets: simple stacking, averaging the overlapped volumes, and a 3D‐3D image registration with the three translational degrees of freedom. Using thermoluminescent dosimeters and custom‐designed holders for a CTDI phantom set, dose measurements were carried out to assess the resultant imaging dose of the technique and its geometric distribution. Deformable registration was tested on patient images generated with the double‐orbit protocol, using both the planning FBCT and the artificially deformed CBCT as source images. The protocol was validated on phantoms and has been employed clinically for IRB‐approved IGART studies for head and neck and prostate cancer patients.

PACS number: 87.57.nj

## I. INTRODUCTION

Since becoming commercially available, LINAC‐mounted cone‐beam computed tomography (CBCT) has gained increasing popularity in clinical settings for image‐guided radiation therapy (IGRT).^(^ ^1^ ^)^ In addition to the radiographic and fluoroscopic imaging capabilities, the kV X‐ray system also enables CBCT acquisition in 3D or even 4D so as to aid in patient positioning, which substantially improves the tumor localization accuracy.^(^ ^2^ ^–^ ^6^ ^)^ At the cutting edge of the field, CBCT also plays a role in many more advanced applications. For example, deformable image registration was used to correlate anatomies between serial CBCT and the planning CT images;^(^ ^7^ ^–^ ^13^ ^)^ dose reconstruction was performed on CBCT images to assess dose consequence of motion or setup errors;^(^ ^14^ ^–^ ^16^ ^)^ and CBCT images were utilized to perform image‐guided adaptive radiation therapy (IGART).^(^ ^17^ ^–^ ^19^ ^)^

**Figure 1:**
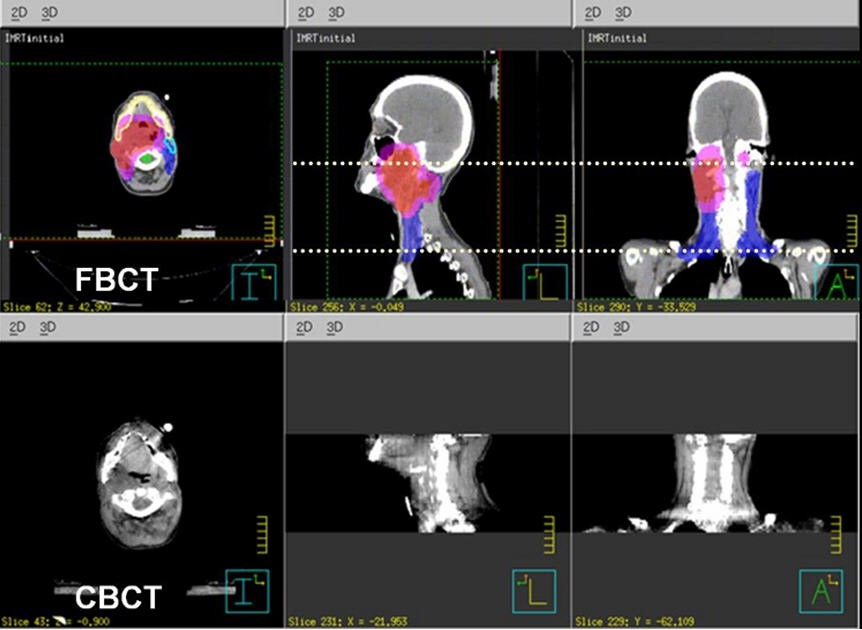
An example where the targets, shown in watershed on the planning FBCT (a), go beyond the maximum longitudinal coverage of the CBCT (b).

These tasks often require an image volume that encompasses the whole treatment field. On the other hand, the size of the flat‐panel detector (FPD) imposes a limit on the image volume in both transverse and longitudinal planes, based on simple trigonometry. For example, the PaxScan 4030CB detector of the On‐Board Imager (OBI) system by Varian Medical Systems, a 40 cm by 30 cm FPD, gives 27 cm and 20 cm maximal theoretical reconstruction dimensions in transverse and longitudinal planes. To extend the imaging coverage in the transverse plane, a “half‐fan” imaging geometry in which the FPD is laterally displaced to project slightly over half of the field of view (FOV) was introduced, extending the transverse FOV up to 48 cm.^(^ ^20^ ^–^ ^22^ ^)^ While this solves the problem for CBCT localization problems by providing the full transverse cross sections for most patients, the longitudinal coverage is still limited to about 14 cm and 16 cm with reconstruction software versions OBI 1.3 and 1.4, respectively. This image length is often shorter than the total treatment field lengths, such as in typical head and neck or prostate treatment fields where nodal coverage is needed. Figure 1 shows the planning FBCT and the CBCT of an example head and neck patient case. The planning target volumes (PTVs) displayed in color watershed go beyond the CBCT longitudinal FOV, with the treatment field and dose calculation box even more substantially beyond it. In this work, we report a technique that is easily implementable by the users to address the longitudinal coverage issue, along with image quality evaluations and dose assessments.

## II. MATERIALS AND METHODS

### A. Image acquisition

In our institution, the On‐Board Imager (OBI) systems (Varian Medical Systems, Palo Alto, CA) are used for CBCT imaging. Software version OBI 1.3 was used at the time of protocol development, which consisted of a single‐rotation scan acquiring about 630 projections over 360°. Two bowtie filters (BTFs) can be used, a full‐fan (FF) BTF for transverse FOV up to 25 cm, and a half‐fan (HF) BTF with a laterally displaced detector for extended transverse FOV up to 45 cm. The reconstructed image lengths of the FFBTF and HFBTF acquisition modes were about 16 cm and 14 cm, respectively. There were two dose levels available, a standard dose mode with the technique factors of 125 kVp, 80 mA, and 25 ms, and a low‐dose mode with those of 125 kVp, 40 mA, and 10 ms. To extend the longitudinal coverage, we proposed a protocol that acquired two consecutive CBCTs with a longitudinal couch translation. The translation was selected to be 14.4 cm with FFBTF and 12.3 cm with HFBTF. This allowed five overlapping slices between the two image sets when acquiring with 3 mm slice thickness. This small overlap was reserved for handling possible misalignment between the two image subsets. The image sets were generated as separate DICOM volumes by the vendor software.

The double orbit acquisition protocol was also later adapted for the upgraded vendor software version OBI1.4. The main change in OBI1.4 was the addition of more diverse acquisition modes better tailored to different imaging tasks with reduced imaging doses. The improved reconstruction of OBI1.4 also increased the length of the reconstructed volumes to about 16 cm for HFBTF acquisitions. Our protocol was hence adapted to have 14.4 cm couch translation to still allow the same amount of overlap.

**Figure 2:**
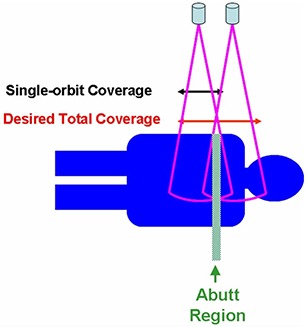
A schematic of the double orbit CBCT acquisition showing the two orbits and the overlap.

Figure 2 shows a schematic drawing of the acquisition geometry. The couch shift was chosen to allow a small overlap between reconstructed image volumes from the two orbits. The overlap was reserved to investigate and evaluate the methods for handling possible misalignments between the two image volumes.

### B. DICOM objects handling

Following acquisition, two separate DICOM image sets would result from the vendor software. To employ the DICOM transfer of commercial treatment planning or image viewing systems, an in‐house DICOM handler was developed using C++ following the description of the DICOM format by Riddle et al.^(^ ^23^ ^)^ The handler reads in the two DICOM sets, reprocesses the header item tags (UIDs (0002, 0003) and (0008, 0018), image number (0020, 0013), and image position (0020, 0032)), and writes the images out into one single DICOM set.

### C. Misalignment handling

In combining the two image subsets into one, three approaches were explored to handle the possible misalignment between the sets. The first way was to simply stack the two image sets using the nominal slice longitudinal position. For the overlapping region, only images from one dataset were kept in the final combined set. In our case we used the images from the superior set. The second way was to average the images from both sets and use the resultant slices for the overlap. The third approach was to realign the two image sets using a 3D‐to‐3D rigid registration. The registration used the mean‐squared difference (MSD) objective function and the regular step gradient descent (RSGD) optimization as implemented by the open source software package Insight Segmentation and Registration Toolkit (ITK) (www.itk.org).^(^ ^24^ ^)^ The MSD is a widely used similarity measure for registering images acquired with the same modality. It calculates the average sum‐of‐square differences of the gray levels between corresponding voxels. The RSGD algorithm advances transformation parameters in the direction of the objective function gradient and a bipartition scheme is used to compute the step size. The registration computed only the three translational degrees of freedom. The calculated translational transformation was then applied on to one image set to realign with the other set by rescaling and reslicing the images. The three methods were applied onto both phantom and patient image sets and compared. A Catphan 600 phantom (the Phantom Laboratory, Salem, NY) and an anthropomorphic phantom in the pelvic region (Humanoid Systems, Carson, CA) were used for the study. On the Catphan phantom test case, a 2 mm shift along the longitudinal direction was intentionally introduced when translating the couch for the second orbit. The phantom was positioned so that the overlapping slice was in the CTP 404 module of the phantom. The CTP 404 module has rod inserts of known Hounsfield numbers that go parallel to the longitudinal axis, and four 23° wire ramps. These wire ramps are used to estimate slice thickness measurements and detect misalignment errors.

### D. Dose assessments

**Figure 3:**
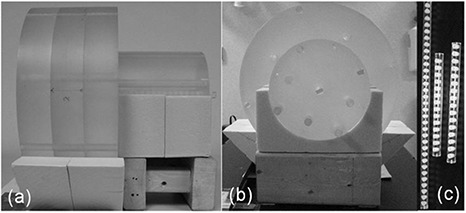
The front (a) and side (b) views of the TLD dose profile measurement setup, and the custom‐made TLD holders (c).

Dose measurements were performed with the computed tomography dose index (CTDI) head and body phantoms.^(^ ^25^ ^)^ The two phantoms were positioned in a way that emulated a typical head and neck patient setup, as shown in Figs. 3(a) and (b). The 16 cm diameter head phantom and the 32 cm diameter body phantom each has a central channel, four peripheral channels, and four intermediate channels, for insertion of either 10 cm pencil ion chambers or our custom‐made TLD holders. The central channels of the two phantoms were aligned in our measurement setup. Both central and peripheral dose profiles were measured. The geometric profile assessment of the imaging dose was carried out using thermoluminescent dosimeters (TLDs) and read out with a Harshaw 3500 TLD reader (Thermo Scientific, Franklin, MA).^(^ ^26^ ^,^ ^27^ ^)^ Customized acrylic holders, shown in Fig. 3(c), were fabricated to hold the TLD chips and fit in the CTDI phantom channels. The holders were machined to hold TLD chips at measurement positions with 1 cm spacing along the axis, and four TLD chips can be stacked at each measurement position. TLD100 chips (3.2 mm×3.2 mm×0.38 mm; ThermoFisher Scientific, Franklin, MA) were used for the study, and our clinical annealing and readout procedure was followed. Dose measurements used OBI1.3 standard dose scans at 125 kVp, 80 mA, and 25 ms for the higher signal level. Four repeated dual‐orbit scans were performed for the measurement to reduce the relative measurement noise and renormalized afterwards. Readings from four stacked TLD chips were taken at each geometric point to assess the statistics of the measurement results. Absolute dosimetry was established by cross‐calibrating the TLD readings in the kilovoltage beam with measurements made using a $0.6\;{\text{cm}}^{3}$ Farmer‐type ionization chamber (PTW N23333; PTW, Freiburg, Germany), with the ADCL‐calibrated kilovoltage air kerma calibration factor $N_{k}$, following the AAPM report TG 61.^(^ ^28^ ^)^ The Farmer‐type chamber was also cross‐compared with a 10 cm pencil chamber (Model 10times6‐3CT, Radcal, Monrovia, CA) in the actual CBCT beam, using their corresponding calibration factors. The agreement was found to be within 2% after accounting for the volume averaging effect. While the TLD measurement was used for investigating the geometric profile of our proposed double orbit protocol, the dose effect by selecting different technique settings (kVp and mAs) was studied by using the 10 cm pencil chamber following standard CTDI measurement procedure, and calculating the weighted CTDI.

### E. Deformable registration test

Once established and validated on phantoms, our double orbit acquisition and processing protocol has been applied when necessary for patients enrolled in two ongoing IGART studies approved by the local IRB. The two studies are for head and neck cancer and prostate cancer patients, respectively, both treated with definitive IMRT. On an example head and neck patient, preliminary deformable registration tests were carried out between the planning FBCT and combined CBCT images, and between the combined CBCT and its artificially deformed counterpart. The artificial deformation applied for the test was Gaussian in the X (lateral) direction, with maximum amplitude of 2.0 cm and width sigma of 12% of the full image length.

The deformable registration used Christensen's inverse‐consistent deformable registration constrained by linear elasticity.^(^ ^29^ ^)^ This algorithm is image intensity‐driven, and it imposes inverse‐consistency by jointly estimating the forward and reverse transformations between two images, while constraining these transforms to be inverses of one another. The test registrations were evaluated qualitatively by visualization of the image overlay, and quantitatively by calculating the cross‐correlation coefficients between the source and target images following reference.^(^ ^30^ ^)^

## III. RESULTS

### A. Extended longitudinal coverage

**Figure 4:**
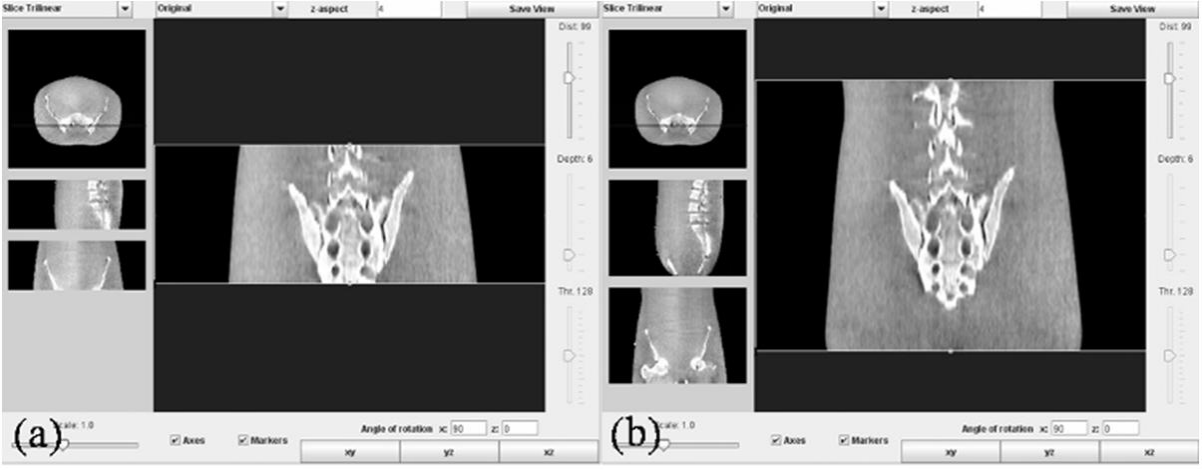
Images from the single orbit scan (a) and the double orbit scan (b) showing the extended longitudinal coverage with the current protocol on a pelvis phantom.

**Figure 5:**
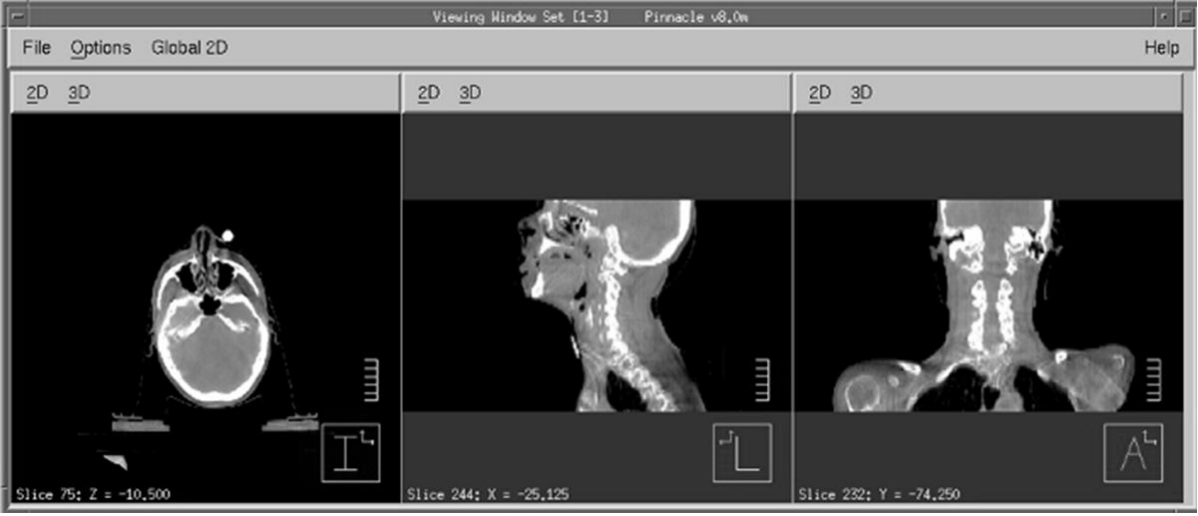
Combined double orbit CBCT images of an example head and neck patient imported into Pinnacle via DICOM.

The image acquisition and processing protocol successfully extended the longitudinal coverage using the commercial CBCT systems. With OBI 1.3, the length of images was extended from 13.8 cm to 26.1 cm with the HF acquisition mode, and from 15.9 cm to 31.8 cm with the FF acquisition mode. Figure 4 shows the extended coverage using our protocol on the pelvis phantom. The resultant image set is one single DICOM volume importable to commercial treatment planning systems. Figure 5 shows the axial, sagittal, and coronal views of an example head and neck patient, acquired with the double orbit HF mode and imported into ADAC Pinnacle (Philips Medical Systems, Milpitas, CA).

### B. Misalignment handling

Misalignment of the images from the two orbits was observed for all patient datasets we acquired, to varying degrees. It was even noticeable on some of the phantom scans, most possibly due to couch sag. On the other hand, the majority of patient images exhibited only small misalignments.

**Figure 6:**
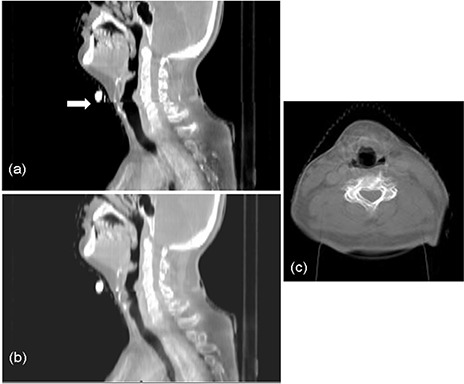
Example patient images showing the efficacy of misalignment handling with the direct stacking (a), rigid registration (b), and averaging (c) approaches.

The three approaches were employed to process all the images. The rigid registration approach always yielded the best aligned image sets, and the averaging approach created obvious blur especially where the original misalignment was large. Figure 6 shows example patient image sets after applying each of the three approaches. From the sagittal images, the misalignment was apparent for the direct stacking approach (6(a)), and better aligned for the rigid registration approach (6(b)). The axial view in Fig. 6(c) shows the blurring effect with the averaging approach.

**Figure 7:**
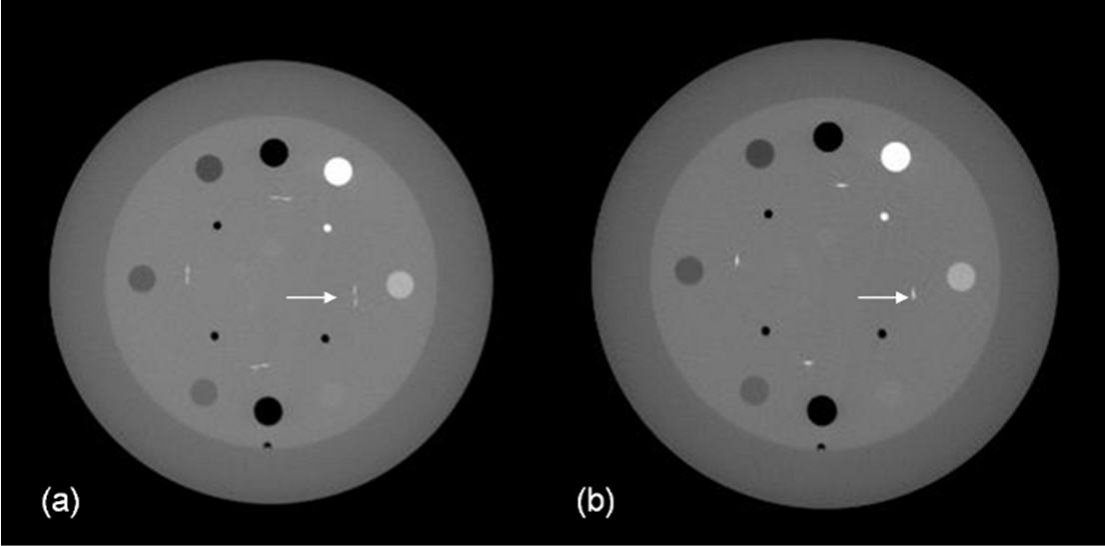
Axial images of the Catphan phantom imaged with intentional couch shift inaccuracy combined with the averaging (a) and rigid registration (b) approaches.

Figure 7 shows the axial images of the Catphan phantom with the averaging and the rigid registration approaches, when a 2 mm couch longitudinal shift error was intentionally introduced during acquisition. With the averaging approach, the axial slice was generated by averaging the two slices from both image subsets in the overlap region that corresponds to the same nominal longitudinal location. As shown in the averaged image, two slightly shifted wires are seen at the position of each wire ramp, indicating a longitudinal misalignment. This could be due to the intentional 2 mm compounded with any intrinsic couch shift inaccuracy. With the rigid registration approach, we first applied rigid registration on the two image subsets; subsequently, the axial image shown was an average of the realigned slices from two subsets that corresponded to the same longitudinal position. The ramp wires from the two image subsets now superimpose on each other, indicating a good longitudinal alignment.

From the patient and phantom tests, the rigid registration approach was the best for handling the image misalignment. When the original misalignment is small, as was the case for most of our patient cases, the direct stacking approach can be a simple way that also provides reasonable image quality. The averaging approach blurs the axial slices significantly where there is misalignment, and should, therefore, be avoided.

### C. Dose profiles

**Figure 8:**
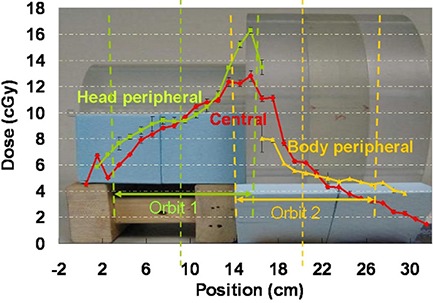
Central channel and peripheral channel dose profiles overlaid with the imaging geometry. The figure shows the dose measured using the OBI 1.3 standard dose scans. For the OBI 1.3 low‐dose scans and the OBI 1.4 low‐dose thorax scans that we have selected for our head and neck protocol patients, the dose should be scaled to about 1/5 and 1/10, respectively.

TLD dose measurements on a phantom setup emulating a head and neck patient using the head and body CTDI phantoms yielded measured dose profiles, shown in Fig. 8. As expected, dose was seen elevating continuously towards the abut region, with the highest dose in the abut region due to the beam overlap. Notably this beam overlap region was wider than the 1.5 cm image overlap, which was because the beam collimation and the cone‐beam geometry went beyond the limits of the reconstructed volumes. Even outside of the area where the primary beams overlap, the dose was higher as it went closer to the abut region, because of increasing scatter. The results shown in Fig. 8 reflected measurements using the 125 kVp, 25 mA, and 80 ms standard dose scan from OBI1.3. The low‐dose scan from the same software version imposed an imaging dose one‐fifth of that from the standard dose scan. The acquisition techniques used in OBI1.4 further reduced the imaging dose, especially for the head scans, resulting in imaging dose reductions up to about 32 folds from the OBI1.3 standard dose mode, depending on the selection of technique settings. The OBI1.4 scan setting we used for our head and neck protocol patients were estimated to result in an imaging dose about one‐tenth of that shown in our phantom measurement. The selection was based on the compromise between the required image quality and the effort to minimize the imaging dose.

### D. Deformable registration test

**Figure 9:**
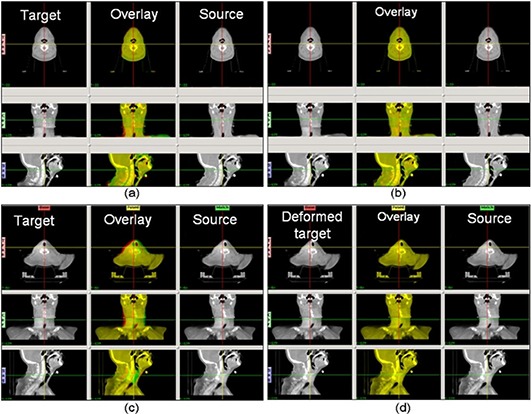
The results from the deformable registration tests. The upper panels show an example head and neck patient CBCT to FBCT deformable image registration with our extended FOV CBCT images: (a) the images before applying the deformable registration, with the target (CBCT) image, the source (FBCT) image, and a color overlay (yellow indicates good agreement, green and red indicate negative and positive disagreement) of the two images; (b) the deformed target, the source, and the overlay images, after applying the deformable registration. The lower panels show the registration between a CBCT and its artificially deformed counterpart, (c) before the registration and (d) after the registration. The CBCT image was used as the target, and the artificially deformed image with a Gaussian was used as the source.

Deformable registration was carried out between patient CBCTs and FBCTs, as well as between patient CBCTs and their artificially deformed counterparts, and the results were shown in Fig. 9. Figure 9(a) and (b) show the results of an example registration of a CBCT to a FBCT. The color overlay demonstrated a successful registration using our double orbit CBCT image. The calculated cross‐correlation coefficients increased from 0.90 before the registration to 0.98 after the registration. Figure 9(c) and (d) shows the results for the test case with the known Gaussian deformation. The deformable registration successfully improved the cross‐correlation coefficients from 0.91 before the registration to 1.00 after the registration. Both cases showed that the images acquired and combined with our protocol were able to support key IGART functions such as deformable image registration.

## IV. DISCUSSION

Misalignment of double‐orbit CBCTs may be due to patient motion, couch sag, and couch shift inaccuracy. While the instantaneous deformation of the patient anatomy, such as patient swallowing or coughing in a head and neck case or gas passing in a pelvis case cannot be easily compensated, a rigid registration helps realign the shifts due to the rigid causes. In the current algorithm, a 3D‐to‐3D rigid registration with only translational degrees of freedom is applied on the abutting overlap region. The overlap was preliminarily chosen to be about 1.5 cm, which was proven to be sufficient for the registration in all our test phantom and patient cases. For clinics that want to implement the acquisition protocol but do not have in‐house rigid registration capabilities, direct stacking can result in reasonable image quality for most patient cases where misalignment is not severe. In this case the overlap can be eliminated, and the couch shift be chosen exactly as the length of one single orbit.

The dose assessment of the imaging protocol showed nearly doubled dose in the abutting overlap region. For the in‐field dose away from the abutting region, the same consideration as in regular single‐orbit scans should be employed to minimize the imaging dose (i.e., to select the most appropriate technique setting, optimizing the imposed imaging dose and the required image quality). On the other hand, it is advisable to reduce the dose to the abutting region in one of a number of ways. In one way, to minimize such high‐dose region, it is advantageous to employ 3D‐to‐2D registration between the partial CBCT volumes and a 2D radiograph covering the abutting region, in which case the overlap can be nearly eliminated. It needs to be pointed out that there will still be increased dose at the abut region even if there is zero overlap, because the cone beam goes beyond the reconstructed image region, as shown in the Fig. 2 schematic. An alternative way for dose reduction using 3D‐to‐3D registration is to reconstruct the shrinking cone volumes at the abut region and apply the registration upon these volumes. This way, the overlap can also be eliminated, if such registration is proven to provide sufficient accuracy. Clinically, it is advisable to estimate the imaging dose throughout the treatment course based on the single procedure dose, imaging frequency, and overall length, especially for the high‐dose region where two volumes abut. If the imaging dose of the high‐dose region adds up to a considerable amount and the dose to the region from the treatment course is already approaching the tolerance of organs at risk, then the abut region can be shifted around from one fraction to another to feather out the increased dose region. Another technique that could be used to avoid unnecessary imaging dose is to set patient‐specific longitudinal beam collimations and adjust the isocenter shift between the two orbits accordingly, to provide no more than necessary image length with the double orbit scan. The collimator can also be set asymmetrically along the longitudinal direction, reducing the beam divergence and the imaging dose to sensitive regions. One example would be a prostate case with nodal coverage in which the inferior orbit isocenter could be set just beneath the inferior border of the field, so that the imaging dose to the gonads will be minimized.

The current protocol only combines the two subvolumes in the image space. Further work could be carried out combining the two scans in the projection space. Potential benefits may arise because the information from each orbit could possibly supplement each other at the abutting region where the large beam angle severely violates the Tuy's sufficiency condition. On the other hand, the current protocol does not require a custom reconstruction, and can be easily implemented at any clinic where the extended longitudinal CBCT coverage is desired.

## V. CONCLUSIONS

An image acquisition and processing protocol was developed to extend the longitudinal coverage of commercial on‐board CBCT images. These images with full coverage of the treatment field could support key tasks of IGART, such as deformable registration and dose reconstruction on serial CBCT images.

## ACKNOWLEDGMENTS

This work was supported by NIH P01 CA116602.
